# An Evacuation Simulation for Hazard Analysis of Isolation at Sea during Passenger Ship Heeling

**DOI:** 10.3390/ijerph17249393

**Published:** 2020-12-15

**Authors:** Imgyu Kim, Hyuncheol Kim, Soonhung Han

**Affiliations:** 1 Department of Mechanical Engineering, KAIST University, Daejeon 34141, Korea; kimimgoo@kaist.ac.kr; 2 i CAPTAIN Co., Daejeon 34052, Korea; ceo@icaptain.kr

**Keywords:** evacuation analysis, isolation at sea, heeling ship, hazard map according to safety factors

## Abstract

The Crisis Management Manual is essential for safe and rapid evacuation in the event of an accident. However, the disaster response measures in the current ship evacuation manual are not systematic and are simple and impractical. In particular, the current instructions when the ship is heeling call for evacuation to the highest deck, in the opposite direction. This advice is dangerous, however, because it is isolated to a specific space, due to the walking limit angle according to the angle of heel during evacuation. This study focuses on the MV Sewol ferry accident on 16 April 2014, to evaluate the risk of isolation at sea when evacuating to the highest deck opposite in the direction of heeling when the ship was sinking. According to the initial angle of heel and various angular velocities, hazard maps were created for each safety factor to predict the risks for each situation, by performing a comprehensive evaluation of the safe and dangerous situations when evacuating to the highest deck. The problems and limitations of the current evacuation manuals and systems were identified, and the necessity of a new evacuation solution was presented.

## 1. Introduction

**Figure 1:**
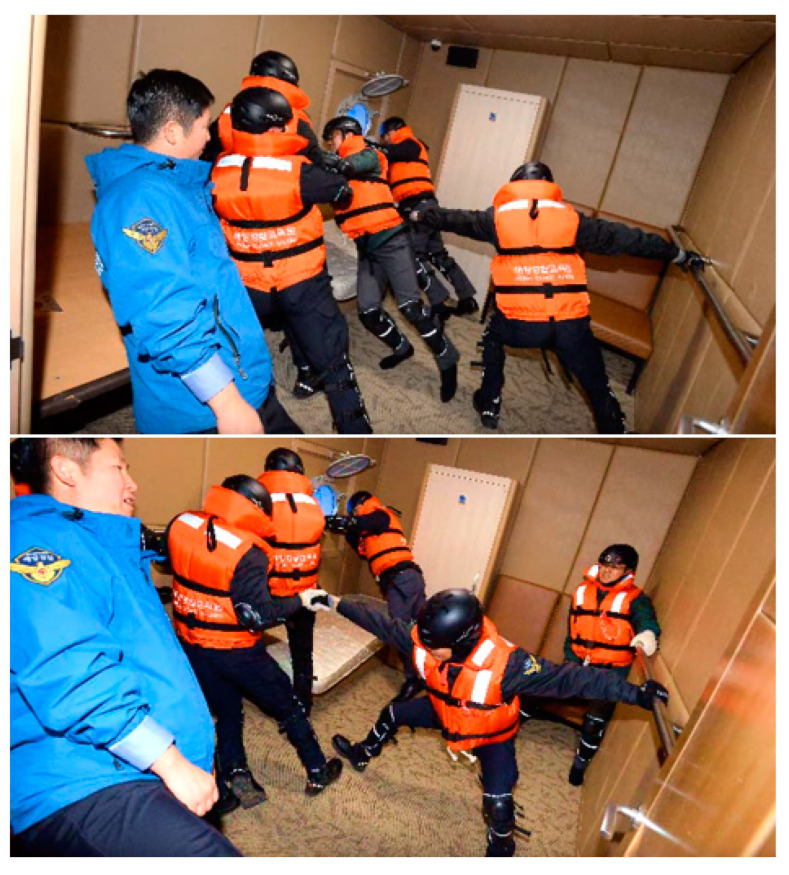
Korea Coast Guard under rescue training on a heeled vessel installed on a motion base [7].

As of 2019, there are over 30 million passenger ships worldwide, representing an increase of 62% over the past decade [1]. As the demand for cruises and passenger ships is expected to continue to increase, it is essential to design vessels that reflect the marine disaster response plan. In general, in the case of a sinking accident, the hydrostatic characteristics change, and the vessel becomes heeled, creating a serious time limit for the evacuation and rescue of the passengers. In addition, due to the nature of passenger ships, paths of movement are narrow and complicated, and the possibility of bottlenecks is very high. In addition, as a sinking ship is an isolated environment at sea, there is great difficulty in rescue, due to the lack of accessibility and safety. Therefore, it is necessary to increase evacuation safety by predicting disaster situations in advance and minimizing risk factors. Internationally, Safety of Life At Sea (SOLAS) [2] and International Maritime Organization Maritime Safety Committee (IMO MSC)/Circ.1533 [3] are prescribed for passenger evacuation safety, and passenger ships must satisfy all of these regulations. SOLAS is an agreement on the safety of life at sea, formed in response to the sinking of the RMS Titanic in 1912, and operators are required to perform a Means of Escape Analysis in accordance with the regulations of SOLAS. Discussion of the analysis of passenger evacuation in IMO MSC began in 1994 with the sinking of the passenger ship MS Estonia, where 852 people died, and evacuation analysis should be conducted in accordance with IMO MSC/Circ.1238 Guidelines on evacuation analysis for new and existing passenger ships. For safe evacuation of the ship, one must not only evacuate to the assembly station but also use lifesaving equipment appropriately. The LSA code defines life jackets and tubes for individual passengers and includes smoke signal and light signal equipment for rescue requests [4]. The most important equipment is the lifeboat and life raft equipment. This includes all passengers and crew members gathering at the embarkation station, boarding according to procedures, and evacuating safely outside the ship. For smaller boats, you can install a life raft instead of a lifeboat. The passenger ship MV Sewol analyzed in this paper was a passenger ship with life-rafts installed, but only one life-raft was launched until it was completely sunk. The International Maritime Organization has rules for lifesaving equipment according to the number of passengers, such as the LSA code, but there is no manual for individual evacuation during the sinking of a ship. Currently, the Korean Marine Vessel Accident Crisis Management Standards Manual specifies that in the event of a disaster, evacuation and rescue requests are requested according to the guidance of the crew [5]. In particular, in the case of the safety manual directions, which call for evacuation to the highest deck on the opposite side when the ship is heeling [6], this does not reflect the risk of isolation at sea, as the walking limit angle changes according to the angle of heel during evacuation. Experiments conducted by the Korea Coast Guard have established that even a healthy adult male finds it difficult to walk at an angle of heel of 30°, and cannot move completely at an angle of heel of 35° [7]. Figure 1 shows evacuation and rescue training while experiencing a scenario of a heeling ship, at a ship disaster training ground built by the Korean Coast Guard on a motion base.

If the passenger ship is sinking in this manner, following the current disaster manual has no effect. In addition, there is no scientific evacuation safety diagnosis and establishment plan for situations where a passenger ship is heeling while sinking. Theories, experiments, and computer-based simulation methods are mainly used to diagnose evacuation safety for passenger ships and establish countermeasures. In the case of theories and experiments, it is possible to analyze only a simple situation. Furthermore, these approaches require a lot of time and money, and for safety reasons, it is very difficult to accurately recreate a disaster environment, making it difficult to predict actual disaster situations. Simulation methods, meanwhile, take a long time to develop, and they can struggle to reflect human characteristics during evacuation. They are very useful, however, because they can analyze various disaster situations that are not possible to address with theoretical or experimental methods, with a systematic engineering approach. In addition, efficient analysis and prediction are possible at a low cost, in terms of time and resources. The analysis of evacuation safety for passenger ships using simulations is recommended by the IMO, and the National Institute of Standards and Technology (NIST) Technical Note 1822 provides examples of evacuation simulations [8]. As such, the effectiveness of evacuation simulations for safety analysis in disaster situations is gradually improving. Here, based on the approach of Kim et al. [9], a study was conducted on the sinking of the passenger ship MV Sewol on 16 April 2014. Simulations were performed at various initial angles and angular velocity conditions to evaluate the risk of isolation at sea when evacuating to a high deck, in the opposite direction the ship was heeling, while sinking. Twelve test cases for software verification of IMO MSC.1/Circ.1533 and verification of SAFEGUARD Validation Data Set 1 [10] were performed [3,9]. For the conditions of all passengers and crew members, the age, gender, and response duration of passengers and crews were distributed according to the criteria of IMO MSC.1/Circ.1533. When moving to a high deck according to the initial angle of heel and angular velocity, a comprehensive evaluation of safe and dangerous situations for isolation was conducted, and a hazard map for each safety factor was created to predict the risk for each situation. In addition, isolated persons were analyzed by gender and age group. Through this, problems and limitations of the current evacuation manuals and systems were identified, and the necessity of a new evacuation solution was presented. This approach permitted the analysis and improvement of the safety of the entire evacuation process for each ship and disaster situation, thus improving the reliability of evacuation safety and reducing social costs in the event of a disaster. In addition, it may be possible to develop effective education and training that can be applied practically in the event of a ship disaster and to minimize damage to life, through rapid and reasonable responses in the event of an actual marine accident, by establishing an optimal evacuation strategy for each situation.

## 2. Problem Definition and Hazard Analysis

In the case of passenger ships, when it hits a reef or receives damage from the outside, a hole is made in the hull shell and the ship is flooded with water. Due to this, the ship’s stability decreases, the center of gravity changes and tilts. As the flood continues, it tilts more and eventually sinks.

The MV SEWOL accident was caused by insufficient stability due to the expansion of the ship, overload of cargo up to two to three times the maximum loading capacity, poor cargo fixation, and less filling of the ballast water that balances the ship. In addition to the exceptionally fast current, all problems acted as a complex cause, causing an accident.

In this study, only the inclination toward the port side was considered by referring to the MV Sewol accident. It is also assumed that the ship is inclined at a constant angular velocity. This is because the angular acceleration is very small when the ship actually sinks, so the change in the angular velocity has very little effect on the passengers and crew.

Before a ship’s inclination reaches 35°, all passengers must travel safely to the assembly station, and those who fail to move are at risk of isolation at sea, increasing their risk of death. In this study, a hazard map was created through evacuation analysis by considering the initial inclination and angular velocity of the ship, so that the crew can quickly respond to accidents by pre-identifying the dangerous angle of heel sections when operating a passenger ship.

A hazard map refers to visualized data that highlight areas that are affected or vulnerable to a specific risk and displays them in a picture form. In general, they are used to prevent major damage by preparing for natural disasters such as earthquakes, volcanoes, landslides, floods, and tsunamis. In this study, based on previous research [9], a hazard map was created for the MV Sewol. Simulations were performed with various initial angles and angular velocity conditions to evaluate the risk of isolation at sea when evacuating to a high deck when the ship is heeled while sinking. The commonalities and differences with previous research [9] are briefly illustrated in Table 1. The effects of crowd density and angle of heel on occupant movement speed were reflected as a reduction factor.

If the hazard map created and analyzed through the simulation of this study is secured for each ship in advance, it can be used when an actual sinking accident occurs. By predicting the isolated personnel according to the initial angle of heel and angular velocity of the ship, the assembly station can be different for each location. An optimal evacuation strategy can be established for each disaster situation.

## 3. Related Works

The design of passenger ships in the 1990s relied on a normative goal-based process. Goal-based design requires ships to be built to a high standard, with the safety of ships in theory being guaranteed by simply complying with regulations and standards. In the 2000s, a philosophical change to ship design occurred, in which attempts were made to analyze and predict risk using a variety of engineering techniques, to improve safety by applying risk-based methods [11]. In a study by Lu and Tseng (2012), important safety evaluation criteria were empirically confirmed to improve passenger ship safety. Safety equipment, ship structure, ship documentation inspection, safety instructions, navigation, communication, and crew members’ ability were presented as the most important items and criteria for safety [12]. Hwang (2013) conducted a walking speed test in a passenger ship for freshmen in unskilled universities [13]. As a result of the experiment, the walking speed decreased by 27.2% due to the ship’s motion due to the voyage of the ship, and the speed reduction in a straight path was greater than that in a cornered path. In addition, as a result of comparing and analyzing the results suggested by IMO, the speed of men and women was faster than IMO standards in “Flat” and “Upstairs,” scenarios, but was slower than those suggested by IMO in “Downstairs” conditions.

Hystad et al., 2016 conducted research on the knowledge and perception of passenger safety and concluded that passengers who have demonstrated and trained on safety have a higher understanding of safety and have greater confidence in the crew [14]. They also stated that it was important to find an effective way to provide information on safety and evacuation.

Vanem and Skjong (2006) proposed a risk-based methodology for using a set of scenarios in evacuation performance evaluation and described a method for deriving a complete evacuation scenario. When using evacuation simulation, the advantages in terms of time and cost are mentioned above, and the advantages of simulation in evacuation analysis have also been described. The potential for the future development of maritime safety regulations has also been discussed [15].

Meyer et al., 2002 identified the issue that evacuation procedures differ depending on the ship in question and argued that it was necessary to apply the same evacuation procedure to all ships [16]. They also highlighted the importance of simulating various disaster situations. Despite the need and importance of various evacuation procedures for ships and disasters, to date, no studies have attempted to establish these safety procedures. One of these reasons is that disaster situations are very complex and dangerous, and it is difficult to explain and reflect various human evacuation characteristics. This means that there are limitations in establishing optimal evacuation procedures for each disaster situation when using existing theoretical and experimental methods.

There are more than 60 evacuation simulation software packages worldwide, the most well-known software packages being FDS+Evac, Finland; Pathfinder, USA; Simulex, England; buildingEXODUS, England; maritimeEXODUS, England; and STEPS, USA [17,18,19,20,21,22].

Currently, most evacuation simulation software packages, studies, and methods focus only on the movement of the occupants and the corresponding evacuation times and do not reflect detailed disaster situations such as the angle of heel of the ship while sinking. They also have limitations in reflecting the characteristics of individual passengers and crowd movements, making it more difficult to analyze evacuation safety more rigorously and accurately. In the case of ships, unlike onshore structures, the angle of heel occurs while sinking, making it difficult to walk. This can significantly reduce passengers’ speed of movement, and increase the total evacuation time.

Therefore, the evacuation simulation of a passenger ship should consider changes in passengers’ moving speed according to the angle of heel. In general, it is known that walking is impossible due to loss of friction when a person encounters an angle of heel inclined to more than 35° [23]. Therefore, all passengers must safely move to the assembly or embarkation station before a ship’s inclination reaches 35°. Rescue may not be possible because unreachable passengers may become isolated by inflowing water. This study is expected to help to build a very effective evacuation system in the case of an actual accident, by understanding the limitations of the current disaster manual and evacuation system and predicting evacuation patterns for each scenario.

## 4. Passenger Ship Evacuation Simulation Considering Heeling

### 4.1. System Architecture

**Figure 2:**
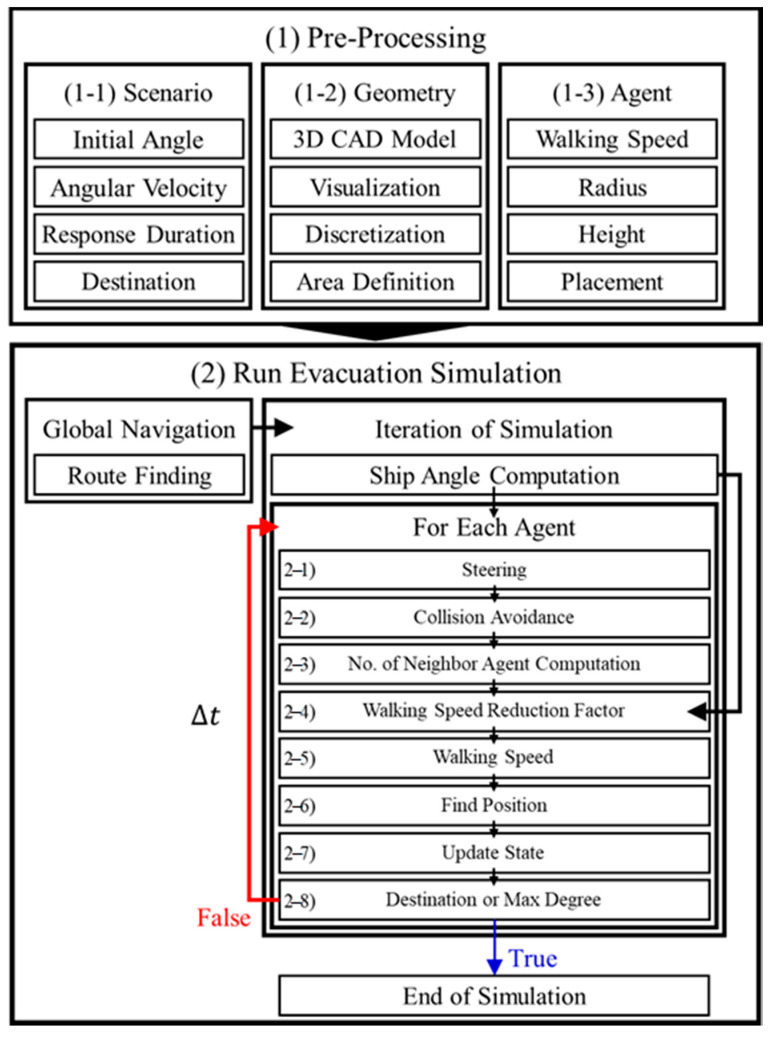
System Architecture.

Figure 2 shows the procedure of the evacuation simulation used in this study.

The simulation comprises a (1) pre-processing stage and (2) an evacuation simulation execution stage. The pre-processing stage is divided into (1–1) Scenario, (1–2) Geometry, and (1–3) Agent.

(1–1) In the scenario stage, the initial angle of heel and angular velocity of the passenger ship are defined. As the moving speed of the occupant is determined according to the initial angle of heel and angular velocity, the initial angle of heel and angular velocity have a direct influence on the total evacuation time and the proportions of isolated and surviving personnel. Response Duration is the time it takes for each passenger to recognize the notification and start evacuation after an evacuation notification occurs. According to IMO regulations, Response Duration was set to from 0 s to 300 s during the day cases and from 400 s to 700 s at night cases; this was logarithmic normal distributed to all occupants shown equation below [3,24].

From night cases:

$$y=\frac{1.01875}{\sqrt{2\pi }0.84\left(x-400\right)}exp\left[-\frac{{\left(\ln\left(x-400\right)-3.95\right)}^{2}}{2\times 0.84^{2}}\right],\;(400<x<700)$$

For day cases:

$$y=\frac{1.00808}{\sqrt{2\pi }0.94x}exp\left[-\frac{{\left(\ln\left(x\right)-3.44\right)}^{2}}{2\times 0.94^{2}}\right],\;(0<x<300)$$

where *x* is the response duration in seconds and *y* is the probability density at response duration *x*.

In addition, in this study, it was assumed that all occupants immediately started evacuating when an evacuation command was issued. This is practically impossible, but a condition in which the Response Duration was set to 0 s was also included in the simulation case.

**Figure 3:**
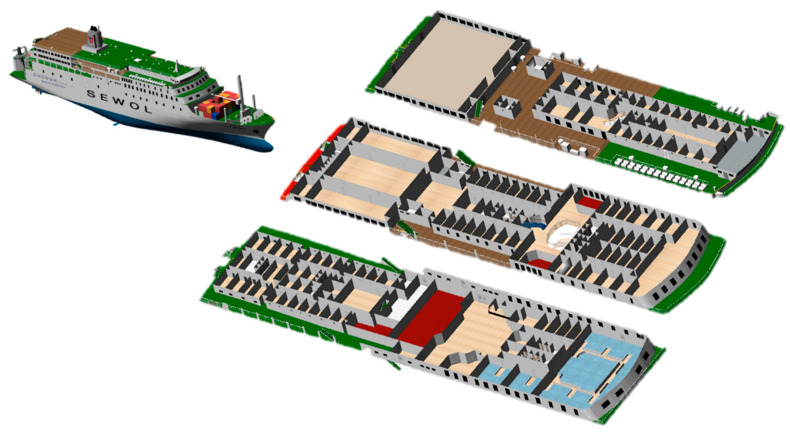
MV Sewol 3D CAD model.

Regarding the total evacuation time, the condition in which the Response Duration was 0 s was the fastest, and the condition in which the Response Duration was 10 min (at night) was the slowest. Destination refers to a place where one occupant can arrive, and also refers to a location that is advantageous to evacuate a safe zone outside by boarding a lifeboat or life-raft. It means the Embarkation station or Assembly station.

(1–2) The geometry module is the area where the simulation is performed, and the movable and non-movable areas must be defined according to the ship’s internal paths, walls, and obstacles. For this definition, the three-dimensional computer-aided design (3D CAD) model of the ship must be secured or created using the dimension information of the drawing. In this study, a 3D CAD model of the Sewol ferry was used as, shown in Figure 3. For the third, fourth, and fifth decks targeted in this study, the movable and non-movable areas were defined in advance.

(1–3) In the agent module, the general characteristics of the occupants were defined, and the minimum and maximum values of the movement speed according to gender and age were randomly distributed to each occupant according to the IMO regulations. The initial locations of all occupants were set to the occupants’ rooms. When starting the simulation, occupants were created in predefined locations.

(1) Once the pre-processing is complete, based on all of the defined information, (2) the evacuation simulation run phase begins. In the global navigation phase, the shortest route to the destination is calculated based on the initial locations of all passengers created. In this study, the A Star (A*) algorithm was used to calculate the shortest path. The A* algorithm a graph/tree search algorithm that finds the shortest path from a given origin to the final destination. It also calculates the cost by evaluating a heuristic estimate to generate the shortest path [25].

When the path creation is complete, the Iteration of Simulation is executed. The simulation iteration is repeated at every step until the end of the simulation. In this study, the time step was set to 0.02 s. If the time step is too small, the simulation run time will be too long. Conversely, if the time step is too large, passengers may break through walls, or passengers may overlap each other. In Iteration of Simulation (2–1) the current ship angle of heel is calculated based on the initial angle and preprocessed angular velocity.

When the ship’s angle of heel calculation is complete, each agent is calculated sequentially, and then moves to the destination. For realistic and viable evacuation simulations, passengers not only have to navigate and find their way but also (2–2) avoid collisions between passengers. In this study, the collision avoidance function between passengers was implemented using the Reciprocal Velocity Obstacles (RVO) model, which was developed to mitigate the agent’s vibration phenomenon, which is a disadvantage of the Velocity Obstacle (VO) model. the RVO model, therefore, provides more realistic evasion behavior to the agent [26].

(2–3) In the Number of Neighbor Agent Computation step, the crowd density is calculated. The travel speed of the occupants depends on the number and availability of other passengers. When there are few other occupants around (i.e., when the crowd density is low), there are no interactions between passengers affecting each other’s movements, so the passengers are not affected when moving and can move at a free walking speed. If other passengers affect their presence and the crowd density is high, the speed of movement of passengers is significantly reduced.

Next, (2–4) the Walking speed reduction factor is calculated by applying both the ship’s angle of heel and the crowd density calculated in advance of the current time. The walking speed reduction factor is one of the key elements of the ship evacuation simulation. Depending on how it is applied, it has a great influence on the overall evacuation time and on the evacuation pattern. We used the speed reduction factor equation derived by performing regression analysis based on the walking experiment results of several research institutes (ETH, ZurichSwiss, KRISO, Monash, SHEBA, SSRC, and TNO) [23]. The calculated walking speed reduction factor is applied to the passengers’ walking speeds given in the preprocessing to calculate the movement speed (2–5) at the current time step. Based on the calculated movement speed, the position to move (2–6) and finally the move (2–7) are calculated.

This process is repeated over and over throughout the simulation; (2–8) the simulation is complete when the passenger reaches the final destination or when the ship’s inclination reaches 35°, that is, the maximum possible travel angle is reached. Before reaching the maximum travel angle of 35°, it was assumed that passengers arriving at the assembly station were safe; passengers who did not reach the assembly station before the angle of heel reached 35° were assumed to be isolated and impossible to rescue.

### 4.2. Simulation Scenario

**Figure 4:**
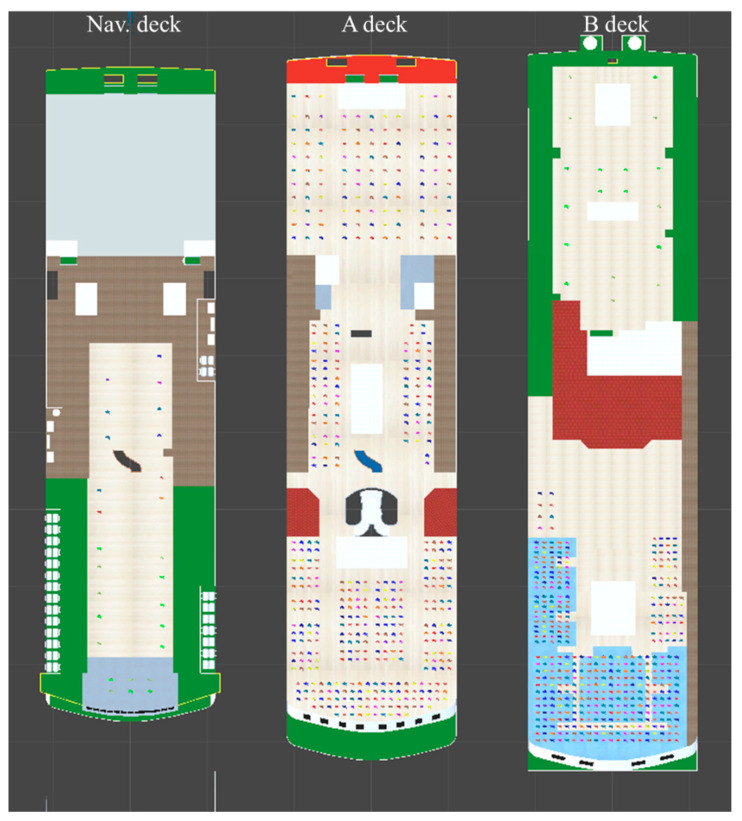
Initial passenger layout. From left, Nav Deck (11 passengers, 16 crew), A deck (484 passengers), and B deck (18 crew, 426 passengers).

In this study, simulations were performed on the third, fourth, and fifth decks (NAV DECK) of MV Sewol, and it was assumed that all passengers were on board (Figure 4). For passengers and crew on each deck, as shown in Figure 4, there were 27 people (11 passengers, 16 crew members) on the fifth deck, 484 passengers on the fourth deck, and 444 people on the third deck (18 crew members, 426 passengers) [27]. The simulation was performed by changing the initial angle, angular velocity, and response time, as shown in Table 1. The actual MV Sewol accident started at an initial angle of heel of 30° and tilted at an angular velocity of approximately 0.5°/min, but in this study, the initial angle and angular velocity were diversified across several values. The initial angle was performed for seven different angles (0°, 5°, 10°, 15°, 20°, 25°, and 30°) in 5° increments from 0 to 30°. The angular velocity was increased from 0.0 to 1.0°/min in 0.25 intervals, a total of five values (0.0, 0.25, 0.50, 0.75, 1.0) were simulated.

In addition to the daytime (300 s) and nighttime (600 s) situations according to the IMO regulations, the passenger’s Response Duration was performed for a total of three cases up to the Response Duration of 0 s. At the time of the MV Sewol ferry accident, an angle of heel occurred in the port direction, and the assembly station was defined as the highest deck in the starboard direction (opposite to the direction in which the ship was inclined). Table 2 shows the available safe escape time (ASET) [28] as a function of the initial angle of heel and the angular velocity for all simulation cases performed in this study.

In all scenarios, the safety of the passenger is guaranteed only if the Required Safe Egress Time (RSET) is less than the ASET. Therefore, when RSET > ASET, this means that there is a risk of maritime isolation, which would mean that passenger safety cannot be guaranteed.

**Figure 5:**
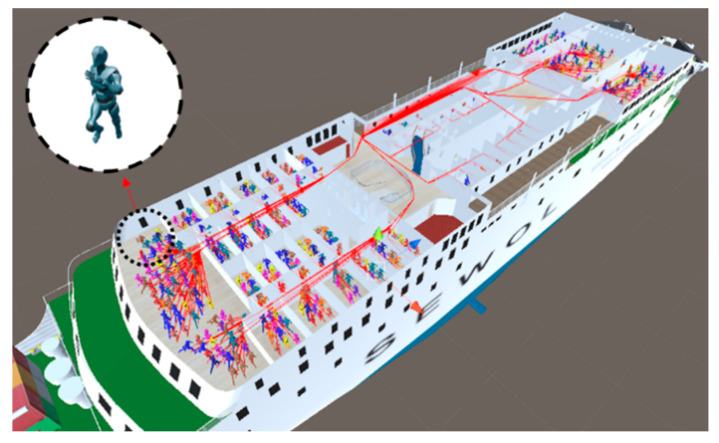
Example of evacuation simulation visualization results.

As Total Evacuation Time (TET) is the most important result in evacuation analysis according to a disaster situation, a total of 50 simulations were repeated with the TET Criterion defined as 0.1%. It was confirmed that the simulation result satisfied the criteria of the convergence measure of TET [29]. An example of the simulation results, created by implementing the system, including the module shown in Figure 2, is shown in Figure 5. The simulation conditions in Figure 5. were performed assuming that the response duration was set as a day case, the initial angle of heel was 30°, the angular velocity was 0.5°/min, and the accident time was 10 am. This is the same as the situation of the MV Sewol ferry accident. Through the visualization results, it is possible to check the interior of the cabin of each deck and the locations of the passengers who are escaping over time. The simulation video for the entire case can be viewed in the Video S1.

## 5. Isolated Hazard Analysis

In the event of a passenger ship sinking, different measures are required depending on the type and characteristics of the accident, and a preliminary prediction is required to establish a strategy for this. An evacuation simulation was performed for each deck of MV Sewol according to the cases in Table 2. Based on the results, the risk according to the occurrence of isolated people was calculated as shown in Table 3.

The percentage indicated below the dot represents the percentage of the total number of people of each deck who had been evacuated, and each number except dot-cells represents the total assembly time (TAT).

If the initial angle of heel is small, the ASET until the angle of heel reaches 35° is relatively large, even if the angular velocity is high, there are no isolated personnel, and all can move to the assembly station. However, with an initial angle of heel of 25°, the difference between the ASET and the last escaped TAT was not significant. This means that even if all personnel successfully escape in the simulation, there is a high risk of containment if an accident occurs under the same conditions. With an initial angle of heel of 25° and an angular velocity of 0.75°/min, isolated personnel occur on deck A and deck B, excluding the NAV deck.

The larger the initial angle of heel and the faster the angular velocity, the more isolated personnel can be predicted through simulation. In a situation where the initial angle of heel is 30°, isolated personnel occur in the NAV Deck, A Deck, and B Deck, and it appears that more personnel are isolated than when the initial angle of heel is 25°. There are more isolated personnel than those who have successfully escaped.

**Figure 6:**
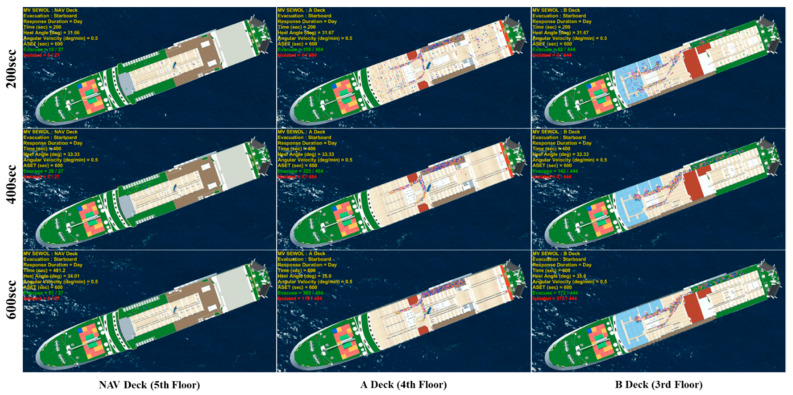
Case No. 33 (initial angle 30°, angular velocity 0.5°/min) simulation visualization result. The entire case can be checked through Video S1.

For Deck B, there are more people in isolation than for Deck A. This is due to the internal shape of the ship itself, and it is judged that the escape point of Deck B is relatively farther away than that of deck A. In the case of the MV Sewol ferry accident, the initial angle of heel at the time of the accident was 30°, and the angular velocity was known to be 0.5°/min (Case 33, Figure 6). As a result of the simulation for the same accident situation as MV Sewol, the simulation of this study predicted that almost half of the people would be isolated even if an evacuation order was issued quickly.

The hazard map generated through this study is an ideal case, and no safety factor was applied for evacuation time (Table 3). In general, a safety factor should be applied to all engineering fields and safety fields, and if a safety factor of 1.5 is applied to the TAT of the generated risk, even if the initial angle of heel is 20°, if the angular velocity is large, then isolated personnel will occur. For a safety factor of 2.0, it was confirmed that even when the initial angle of heel was 15°, the angular velocity was large, and in the case of nighttime, isolated personnel occurred.

**Figure 7:**
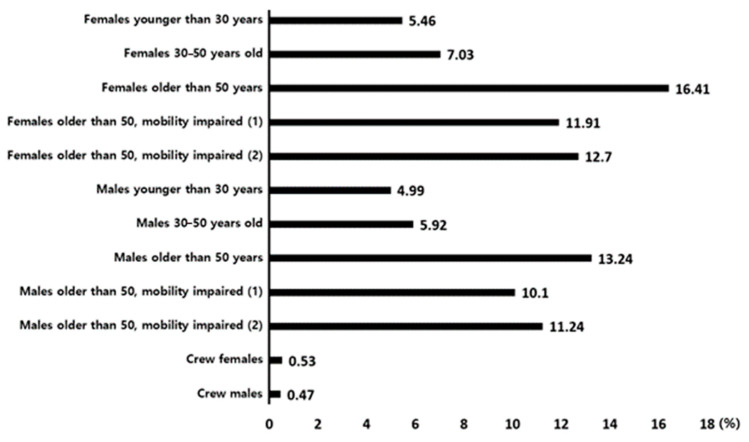
Isolated person rate according to population composition (age and gender).

In the simulation, the ratio of gender and age distribution of passengers and crew members was applied according to the criteria of Table 3.1. Population composition (age and gender) of IMO’s MSC.1/Circ.1533 [11]. Figure 7 shows the results of the distribution of passengers and crew by gender and age of isolated personnel. The numbers shown in Figure 7 represent the ratio of the population to the sum of the number of isolated persons that occurred in the cases indicated by dots in Table 3.

For Crew, less than 1% of the population was isolated, for both males and females. The reason for the relatively small number of isolated crews is not only that the number of passengers aboard the passenger ship is relatively small, but also that the movement speed of the male crew according to the IMO standard is equal to “Males younger than 30 years,” and the movement speed of the female crew is given the same speed as “Females younger than 30 years.” That is, it is predicted as the result shown because their moving speed is relatively fast.

Overall, it can be seen that both men and women are less isolated when they are young. According to the IMO standard, the younger the person moving, the faster their movement speed. Therefore, most personnel can easily evacuate before reaching the maximum angle of 35°.

In the case of women, the largest number of isolated people occurred in the “Females older than 50 years” group. In this group, the movement speed itself is faster than the “Females older than 50, mobility impaired (1)” and “Females older than 50, mobility impaired (2)” groups. However, according to IMO Population’s composition regulations, “Females older than 50 years” accounts for 16% of the total population, whereas “Females older than 50, mobility impaired (1)” and “Females older than 50, mobility impaired (2)” must each account for 10% of the total. Therefore, despite their relatively high speed, it is predicted that the largest number of isolated personnel occurred for “Females older than 50 years” because more personnel would be on board.

In the case of men, the highest number of isolated people occurred in “Males older than 50 years,” as in the case of women. This is judged to be the result of the same reason as the Female group. Analyzing the simulation by gender and age for the number of isolated people reveals that the ratio of the number of isolated people seems to be directly related to moving speed. In the case of simulation, the physical ability of passengers and crew members is represented by movement speed, but in the case of an actual accident, for higher physical ability (including physical strength), a smaller probability of isolation would be expected.

If a hazard map, created and analyzed through the simulation presented in this study, is secured for each ship in advance, it is possible to establish various evacuation strategies according to the initial angle of heel and angular velocity of the ship in case of an actual sinking accident. By predicting isolated personnel, assembly stations can be set differently for each deck, and evacuation strategies can be adaptively established according to the disaster situation. This approach is expected to minimize human damage and maximize evacuation safety.

## 6. Conclusions

In this study, a simulation was performed that reflected changes in the occupants’ moving speeds according to the inclination of the ship. The risk of isolation at sea was analyzed and predicted according to the initial angle of heel and angular velocity of the ship. In addition, there is a need for different evacuation procedures and types for each ship and for each disaster situation. Simulations to predict the disaster situation for this purpose, the need was mentioned.

ASET (Available Safe Egress Time) varies according to the simulated initial angle of heel and angular velocity. The simulated time taken to move to the assembly station can be compared with the ASET to determine the success of the evacuation. This information can then be used to determine if other evacuation measures have to be carried out.

The hazard map derived through this study is expected to help the captain and crew make decisions about whether to evacuate passengers to the highest deck, or whether to put on a life jacket and send it out to sea based on the initial inclination and angular velocity when the ship sinks. In addition, according to the rules of the International Maritime Organization, we currently have lifeboats and life jackets that can be used by all persons on board. However, in certain cases when the ship is sunk, lifesaving equipment may not be available due to time and distance limitations. If life-saving equipment is placed in consideration of this in the design stage, it will lead to more safe and efficient evacuation.

The hazard map derived in this study has limitations in that visibility and intuition are somewhat insufficient. In future work, research that can derive an efficient visualization method of multidimensional data will be conducted, and a tool for intuitive decision-making in emergency situations will be proposed. In the future, it should be possible to establish a strategy to minimize the number of isolated people in the event of sinking. In order to efficiently use a hazard map in the event of a real disaster, research should be conducted into smart evacuation systems as well. With advances in artificial intelligence and IoT technology, technologies such as safety guidance lights and active route indicators in case of emergency are under study [30,31]. If these technologies are employed in passenger ships, it is expected that more efficient and safe evacuation will be possible by supporting the decision-making of the captain and crew.

## Figures and Tables

**Table 1:** Technical comparison with previous work.

	Kim et al. [9]	This Work
Considering Angle of Heel	Yes	Yes
Method Considering Angle of Heel	Reduction Factor	Reduction factor
Hazard Analysis During Heeling	No	Yes
Hazard Map	No	Yes
Possibility of Utilization in Case of Actual Ship Accident	No	Yes

**Table 2:** Cases of evacuation simulation.

Case	1	2	3	4	5	6	7
Initial Angle (degree)	0.0	5.0	10.0	15.0	20.0	25.0	30.0
Angular Velocity (degree/min)	0.00 0.25 0.50 0.75 1.00						
ASET (Available Safe Egress Time, s)	Infinity 8400 4200 2800 2100	Infinity 7200 3600 2400 1800	Infinity 6000 3000 2000 1500	Infinity 4800 2400 1600 1200	Infinity 3600 1800 1200 900	Infinity 2400 1200 800 600	Infinity 1200 600 400 300

**Table 3:** Hazard map according to sinking condition.

No	Initial Angle (degree)	Angular Velocity (degree/min)	ASET (s)	NAV Deck (5th Deck)			A Deck (4th Deck)			B Deck (3rd Deck)		
				Response Duration								
				None	Day	Night	None	Day	Night	None	Day	Night
1	0.0	0.00	Infinity	62.4	294.91	610.8	283.31	361.31	653.89	446.72	498.22	677.98
2		0.25	8400	58.8	302.51	633.79	287.91	366.01	667.48	440.12	500.52	710.57
3		0.50	4200	54.8	321.51	603.2	294.11	365.51	655.89	445.62	482.22	674.68
4		0.75	2800	60.2	306.11	637.89	289.01	372.91	659.99	447.32	490.12	686.08
5		1.00	2100	58.3	341.21	630.69	289.01	381.61	660.19	444.32	500.42	651.49
6	5.0	0.00	Infinity	78.6	321.81	605.3	287.81	373.01	646.49	451.22	473.52	674.38
7		0.25	7200	79.4	334.41	603.4	290.11	365.41	673.68	430.42	506.22	681.98
8		0.50	3600	49	353.41	605.8	281.61	370.71	692.08	449.22	489.02	673.68
9		0.75	2400	68.3	336.01	610.3	290.61	366.01	638.29	450.92	514.52	686.18
10		1.00	1800	66.4	307.51	635.99	297.81	359.41	663.19	467.82	507.72	672.18
11	10.0	0.00	Infinity	64	302.71	611.8	313.41	354.31	650.99	469.72	513.72	693.18
12		0.25	6000	68	304.31	597.3	300.11	357.71	649.49	456.02	494.72	680.18
13		0.50	3000	57.9	327.01	617.9	291.31	361.81	651.69	464.22	502.02	721.37
14		0.75	2000	68.1	351.41	597.8	289.81	360.21	668.68	473.32	521.02	676.68
15		1.00	1500	56.6	346.41	609.7	301.31	380.31	654.09	463.12	486.22	668.98
16	15.0	0.00	Infinity	59.5	304.11	565.91	292.31	364.21	699.38	471.72	512.22	680.28
17		0.25	4800	73.6	363.71	603	298.41	367.61	664.28	469.82	516.32	708.97
18		0.50	2400	91.3	310.41	575.61	303.41	394.81	683.08	482.12	528.72	702.18
19		0.75	1600	74.2	328.51	679.48	313.81	368.01	668.18	468.32	552.91	699.78
20		1.00	1200	103.3	319.61	586.3	306.31	383.91	663.49	483.62	550.81	697.28
21	20.0	0.00	Infinity	56.6	380.11	653.29	317.61	378.41	671.58	493.72	543.61	692.68
22		0.25	3600	61.9	301.81	657.39	324.11	373.71	690.88	519.22	536.72	687.28
23		0.50	1800	93.3	311.71	616.6	333.61	397.12	679.88	517.82	576.21	704.68
24		0.75	1200	69.7	340.41	604.1	317.91	382.51	705.97	556.31	586.6	745.27
25		1.00	900	83.7	361.1	591.4	338.11	401.92	722.97	564.81	623.69	822.95
26	25.0	0.00	Infinity	98.0	348.6	609.02	352.11	401.72	675.08	551.91	602.1	706.27
27		0.25	2400	82.2	314.4	573.42	363.51	417.12	669.48	586.7	653.39	716.47
28		0.50	1200	93.6	356.0	628.22	383.31	426.32	713.17	656.19	730.47	836.54
29		0.75	800	63.4	345.0	594.9	399.52	486.92	● 95.5%	● 96.4%	● 85.6%	● 77.9%
30		1.00	600	103.0	317.0	● 74.1%	461.62	● 98.8%	● 73.1%	● 75.9%	● 62.8%	● 52.5%
31	30.0	0.00	Infinity	149.4	310.0	675.02	505.22	535.92	706.37	832.54	871.03	928.42
32		0.25	1200	140.6	384.4	698.42	662.19	735.07	881.23	● 89.6%	● 82.4%	● 72.1%
33		0.50	600	146.4	481.2	● 70.4%	● 78.9%	● 75.4%	● 52.9%	● 46.4%	● 38.7%	● 32.0%
34		0.75	400	144.0	● 74.1%	● 48.1%	● 65.1%	● 50.0%	● 28.5%	● 31.8%	● 23.0%	● 17.1%
35		1.00	300	185.8	● 48.1%	● 33.3%	● 53.1%	● 30.8%	● 21.1%	● 24.3%	● 12.8%	● 10.8%

If members cannot evacuate due to isolation, they are marked with black dots (●).

## Supplementary Materials

The following are available online at https://drive.google.com/file/d/15AnqQgW1wlWl-1pZA8TllTmJ-u0wA4Ah/view?usp=drive_web, Video S1: An Evacuation Simulation for hazard analysis of Isolation at sea during passenger ship heeling.
